# LPWAN Key Exchange: A Centralised Lightweight Approach

**DOI:** 10.3390/s22135065

**Published:** 2022-07-05

**Authors:** Gaurav Pathak, Jairo Gutierrez, Akbar Ghobakhlou, Saeed Ur Rehman

**Affiliations:** 1 School of Engineering, Computer and Mathematical Sciences, Auckland University of Technology, Auckland 1142, New Zealand; gaurav.pathak@aut.ac.nz (G.P.); akbar.ghobakhlou@aut.ac.nz (A.G.); 2 College of Science and Engineering, Flinders University, Tonsley, SA 5042, Australia; saeed.rehman@flinders.edu.au

**Keywords:** LPWAN, key exchange, IoT security, session keys

## Abstract

The Internet of Things (IoT) is one of the fastest emerging technologies in the industry. It includes diverse applications with different requirements to provide services to users. Secure, low-powered, and long-range transmissions are some of the most vital requirements in developing IoT applications. IoT uses several communication technologies to fulfill transmission requirements. However, Low Powered Wide Area Networks (LPWAN) transmission standards have been gaining attention because of their exceptional low-powered and long-distance transmission capabilities. The features of LPWAN transmission standards make them a perfect candidate for IoT applications. However, the current LPWAN standards lack state-of-the-art security mechanism s because of the limitations of the IoT devices in energy and computational capacity. Most of the LPWAN standards, such as Sigfox, NB-IoT, and Weightless, use static keys for node authentication and encryption. LoRaWAN is the only LPWAN technology providing session key mechanisms for better security. However, the session key mechanism is vulnerable to replay attacks. In this paper, we propose a centralized lightweight session key mechanism for LPWAN standards using the Blom–Yang key agreement (BYka) mechanism. The security of the session key mechanism is tested using the security verification tool Scyther. In addition, an energy consumption model is implemented on the LoRaWAN protocol using the NS3 simulator to verify the energy depletion in a LoRaWAN node because of the proposed session key mechanisms. The proposed session key is also verified on the Mininet-WiFi emulator for its correctness. The analysis demonstrates that the proposed session key mechanism uses a fewer number of transmissions than the existing session key mechanisms in LPWAN and provides mechanisms against replay attacks that are possible in current LPWAN session key schemes.

## 1. Introduction

With the evolution of wireless communication technologies and mobile computing, numerous novel use cases of network-based applications are evolving. One of the paradigms recently gaining attention is the Internet of Things (IoT). IoT can be described as a network of smart devices at a global scale that provides the facilities to automate the real world through monitoring, data collection, and data analysis [1]. The requirements of IoT applications can vary depending on their requirements. However, two of the most important requirements of IoT applications are energy efficiency and long-range transmission from IoT devices. Low Powered Wide Area Networks (LPWAN) fit perfectly into the energy-efficient long transmission requirement of IoT networks.

LPWAN communication standards are long-range communication technologies using low frequencies for transmissions. These technologies can provide low data rate communication up to a distance of 45 km in rural and 5 km in urban zones using a star topology [2]. LPWAN standards are gaining the attention of the industry as well as academia because of their potential in diverse applications for IoT.

As the popularity of LPWAN technologies grew, multiple vendors joined the competition for providing IoT services with LPWAN communications. Currently, LPWAN standards are available in both licensed and unlicensed frequency bands. Some of the leading LPWAN technologies are Sigfox [3], LoRaWAN [4], NBIoT [5], and Weightless [6].

LPWAN has multiple transmission standards under its umbrella, and all of them are promoted by different vendors. Hence, all LPWAN standards have different transmission and security mechanisms. However, because of the limitation of resources in network nodes, they rely on basic security techniques. LPWAN standards do not implement public-key cryptography or session key mechanisms as nodes cannot carry out any computationally intensive operations at their ends. This limitation of using basic security mechanisms in the network creates security vulnerabilities in the network [7].

Most of the LPWAN communication technologies use a shared secret key for node authentication and data confidentiality [8]. The secret keys are securely stored in the devices before the deployment of the nodes and the same secret key is used for authentication and confidentiality throughout the node lifetime. Using the same key for a long period of time creates a possibility for the attackers to collect enough information for cryptanalysis [9]. Hence, it is recommended to update secret keys or use session keys for enhanced security of the network [10]. However, the implementation of session key mechanisms can cause additional transmissions from end nodes, causing additional overhead on nodes, resulting in a shorter node lifetime [11]. Another option is to use public-key cryptography, where two different keys are used for encryption and decryption of the data. However, public-key cryptography requires extensive computations and is not considered suitable for resource-constrained devices [11].

Amongst all the communication technologies, LoRaWAN is the only LPWAN technology that offers over-the-air session key generation [7]. LoRaWAN provides two-node activation mechanisms, Activation by Personalisation (ABP) and Over-The-Air-Activation (OTAA) [4]. The session key mechanism is only provided by OTAA in the joining procedure of the end node. When the node joins the network, it initializes a join request. Following the join-request, the network server responds with a join-accept message. The node uses the data in the join-accept message to generate application and network session keys. The LoRaWAN session key mechanism is initiated by the end node whenever the node is reset or the frame counter of the node is reset (reaches its maximum value) [4]. It uses random nonce with the join-request message to avoid replay attacks. However, the join-request accepts messages sent from the network server that does not have any replay attack prevention mechanism, creating a possibility of a replay attack on the network [12].

As discussed above in this section, LPWAN is one of the most popular communication technologies for low data rate IoT applications. However, there are several limitations and security vulnerabilities in existing LPWAN technologies. Considering the vulnerabilities of current security mechanisms and the resource limitations of end nodes in the network, a lightweight and secure session key mechanism is required to enhance the LPWAN security without placing an additional burden on end nodes [11,13,14].

This article takes the motivation from the requirements of LPWAN and proposes a lightweight session key mechanism that can be used in any LPWAN communication standard that follows star topology in the network. The proposed framework is validated for its security, effective operations, and energy efficiency during the session key mechanism in a constrained network environment. The proposed session key mechanism focuses majorly on reducing the operational overhead from the end nodes by using lightweight key calculation operations and reducing the number of transceiver operations. This session key mechanism requires only a single transmission from the end nodes for a successful session key calculation.

The remainder of the paper is organized as follows: Section 2 discusses existing key exchange mechanisms in LPWAN and similar networks. Section 3 provides the details of the proposed session key mechanism, the sequence of transmissions, and the mathematical calculations performed to obtain a common session key between network nodes. Further, Section 4 provides the details of experimentation, analysis of the results, and a comparison with existing LPWAN session key mechanisms. Finally, Section 5 concludes the paper and discusses the future direction of the research.

## 2. Related Work

The unavailability of session key mechanisms in resource-constrained devices causes security vulnerabilities in the networks. These devices can be exposed to attacks because of these vulnerabilities when deployed in the IoT networks. As these devices are always connected to the Internet, there can be security breaches causing serious damage to confidentiality, integrity, authenticity, and privacy in the network. The IoT devices can be used as a gateway to launch attacks as they cannot use computationally extensive security mechanisms. To avoid attacks using IoT nodes, there have been numerous attempts involving session keys and key update mechanisms for constrained networks such as LPWAN.

In [15], security vulnerabilities of LoRaWAN are discussed along with possible attacks. It is highlighted that LoRaWAN provides a session key with every new join-request by the devices in the network. However, the keys used to generate the session keys are static and not updated in LoRaWAN. A key update mechanism is proposed to change the static key used for session key generation periodically. A two-step Key Generation Function (KGF) is proposed where both steps use Pseudo-Random Number generation on a Rabbit stream cipher to obtain a key stream. However, it is not explained how this mechanism will scale and how the synchronization of the network server and end nodes will be achieved for key update requests, as end nodes are not always listening for incoming transmissions.

In [16], LoRaWAN key management mechanisms are studied and an alternative technique for session key updates is proposed. The use of Ephemeral Diffie–Hellman Over Concise Binary Object Representation (CBOR) Object Signing and Encryption (COSE) (EDHOC) for session key update is recommended because of its lightweight computations and its limited transmission requirements. A detailed comparison between Internet Key Exchange v2 (IKEv2) [17], Datagram Transport Layer Security (DTLS) [18], and EDHOC based on their key derivation mechanism is made. It is highlighted that DTLS and IKEv2 are not suitable for session key generation in LoRaWAN as they are not designed to work in a highly constrained environment. Hence, the authors have suggested using EDHOC and found it better suited for LoRaWAN devices for enhanced security.

In [11], the authors have discussed the inapplicability of asymmetric key cryptography in constrained devices for key exchange. Considering the device limitations, a seven-step key generation process is proposed for regular key refreshments in LoRaWAN networks. The key agreement involves various operations for each step of the key generation that is performed on the physical layer parameters of LoRaWAN between the gateway and the end device. The authors have performed extensive experiments to demonstrate the accuracy of the proposed key generation method. However, there is no analysis of the energy consumption of the proposed algorithm in the LoRaWAN network. As the proposed mechanism in the paper uses several steps that require the nodes to perform a number of calculations, there is a possibility of node lifetime being shortened.

A physical layer message authentication algorithm is proposed in [19] for node authentication in LPWAN networks. The proposed scheme relies on physical layer parameters and a pre-shared secret key used between devices to generate an authentication code. To collect the physical layer information, the nodes extract channel parameters such as the Channel State Information (CSI) and the Received Signal Strength Indicators (RSSI). The extracted channel information and the pre-shared secret are used to authenticate the nodes in the network. However, the use of channel information for authentication can be limited to static networks. With the mobility in the network nodes, the RSSI will vary, causing challenges in authenticating the nodes.

A mutual device-to-device authentication mechanism with forwarding secrecy is proposed for ZigBee devices [20]. The protocol uses symmetric key encryptions and enables devices to have a key agreement for a session key. The session key is changed frequently to provide forward secrecy. Pre-deployment, every node has a unique ID and a key generated by using devices’ inner circuit chips. This key is considered the secret key for the device. All the devices register themselves to a controller. Access control for all the devices is also configured during device registration. For the device-to-device communications, the nodes use a controller as a middleman to authenticate each other and then come to a session key agreement.

A dual key activation scheme for LoRaWAN is proposed in [21]. This paper discussed loopholes in LoRaWAN node activation mechanisms, such as the use of static keys for session key generation. To address this issue in the node activation mechanism, a six-step activation mechanism for node activation is proposed. The newly introduced approach focuses on using two different keys for generations of network and application session keys rather than using a single key for the generation of all session keys. Once the session keys are generated on both server and node ends, the pre-stored keys used to generate the session keys are discarded and the generated keys are used for all further transactions. However, as nodes stores two keys in the initial key setup for the application and network servers, it can create a requirement for a third-party key management entity as the network scales.

As authentication of nodes became challenging when attackers used the prestored keys to breach the network authentications, Physical Unclonable Functions (PUF) [22] were introduced for node authentication of nodes based on hardware manufacturing irregularities in the nodes. In PUF-based authentications, a challenge is given to the PUF, which is an electronic circuit and based on the response, the node is authenticated. As the manufacturing irregularities of every circuit are said to be unique, the response to challenges is also unique as they are passed through an electronic circuit. However, the use of PUFs can introduce a requirement for additional hardware with the node.

As discussed in the literature, there have been a number of attempts to facilitate stronger security mechanisms for LPWAN-based IoT networks. Different approaches are adopted by researchers to achieve better security for constrained networks like LPWAN. Some approaches use physical layer parameters to identify nodes. However, this introduces additional hardware requirements with the nodes in the network. On the other hand, some of the researchers aim to achieve a session key mechanism introducing additional transmission overhead, which is one of the most energy-demanding operations for end nodes [23].

This research proposes a lightweight key agreement mechanism that aims to provide a session key mechanism while using minimal transmissions and lightweight calculations considering the node’s power consumption. The following sections of the paper discuss the proposed session key mechanism along with its features and performance.

## 3. Proposed Key Exchange Mechanism

Considering the available key exchange mechanisms for LPWAN and similar technologies, there are still challenges regarding the provision of power-efficient, robust and scalable key exchange mechanisms. As discussed in Section 2, the existing techniques for session keys in constrained IoT networks require multiple transceiver operations that can lead to a shorter node lifetime in the network.

In this research, a robust key agreement mechanism for LPWAN technologies is proposed for LPWANs. As most of the LPWAN standards use a star topology, this research leverages the star topology of LPWAN with the Blom–Yang key agreement (BYka) [24] scheme to provide a session key mechanism. The BYka scheme is a key agreement technique for sensor networks where two nodes generate a common key by using node ID as public information. However, in BYka, every time, the same node pair will generate the same key for data encryption and authentication because the pre-stored information in both nodes is static. This research extends the BYka scheme to fit the LPWAN architecture’s characteristics and uses it for session key generation.

### 3.1. The BYka Scheme

The BYka scheme uses Blom’s key agreement mechanism [25] for primitive operations and modifies it by using multiple public keys and master keys for private key generations.

**Figure 1:**
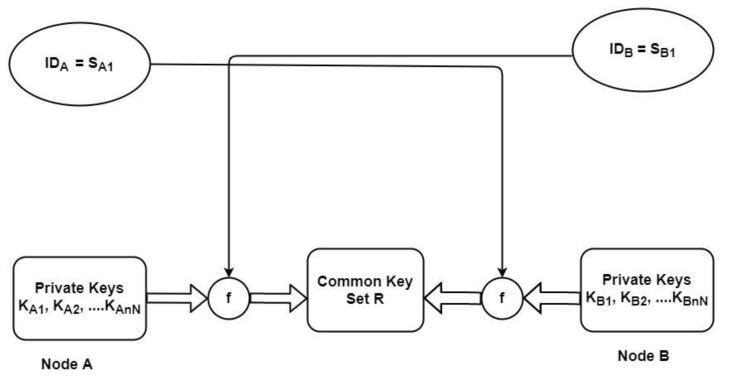
The BYka Process.

Figure 1 shows the process of the key agreement in the BYka scheme. The secret key is calculated using permutations with a private key set of the node; pairwise key set R is calculated in this process:

The algorithm consists of various variables/parameters—*N* is the number of master keys where each key is a random matrix of size *m* × *m*, *n* is the number of public keys, *q* is the prime modulus for the public key, and *p* is the prime modulus for other key operations. The values of *N, m, n, p*, and *q* are considerably small yet enough to provide 128-bit key equivalent security.

Notations used in BYka procedure:

*S: Public key seed value*
*V: Public key; m × 1 column vector*
*R: Set of integers for forming pairwise key between two nodes*
*M: Master key, secret symmetric m × m matrix belongs to Server*
*p: Prime modulus for key operation*
*n: Number of public keys assigned to each node*
*N: Number of master keys*
*q: Prime modulus for public keys*

The public keys of a node are Vandermonde matrices and can be calculated as follows:

(1) $$V_{i}^{T}=\left[1,s_{i},s_{i}^{2},s_{i}^{3},\;\ldots s_{i}^{m-1}\right]mod\;q$$

where *s_i_* = Node ID + *i* − 1, for *i* = 1, 2, …, *n*.

The private key set S = K_11_, …, K_nN_ for every node is a permutation of N master keys and *n* public keys. Private keys for all the node IDs are calculated and securely transferred to every node before its deployment. The private keys for nodes are calculated using the following equation:

(2) $$K_{\mathrm{ij}}\;=\;V_{i}^{T}\;Mj\;\mathrm{mod}\;p$$

for *i* = 1, 2, …, *n* and *j* = 1, 2, …, N.

As shown in Figure 1, each node pair calculates the keys upon receiving public key information from each other. The operations performed by any node pair A and B are as follows:

Node A:

For *i, k* = 1, 2, …, *n* and *j* = 1, 2, …, N

(3) $$\begin{array}{c} s_{Bk}\;=\;ID_{B}\;+\;k-1\\ V_{Bk}^{T}\;=\;[1,\;s_{Bk},\;\ldots s_{Bk}^{m-1}]\\ R_{Aijk}\;=\;K_{Aij}V_{Bk}\;=\;(V_{Ai}^{T}M_{j})V_{Bk}(mod\;p) \end{array}$$

Node B:

For *i, k* = 1, 2, …, *n* and *j* = 1, 2, …, N

(4) $$\begin{array}{c} s_{Ak}\;=\;ID_{A}\;+\;k-1\\ V_{Ak}^{T}\;=\;[1,\;s_{Ak},\;\ldots s_{Ak}^{m-1}]\\ R_{Bijk}\;=\;K_{Bij}V_{Ak}\;=\;(V_{Bi}^{T}M_{j})V_{Ak}(mod\;p) \end{array}$$

After calculation of all the elements of R, elements in R for both nodes are the same, but not in the same order. Hence the secret key can be calculated by either of the following operations on both nodes:

- Multiplying elements of R.
- Sorting the set elements of R.
- Counting of occurrence of an integer in R.

### 3.2. The Session Key Extension to BYka

**Figure 2:**
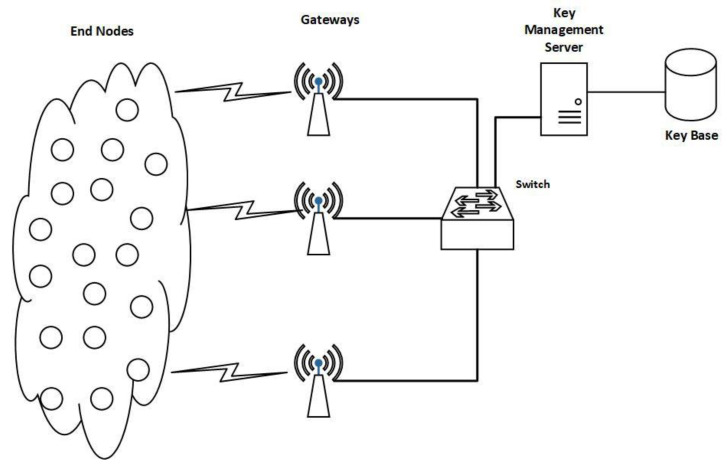
Session Key Architecture.

As discussed in Section 3.1, BYka is an efficient and lightweight key agreement mechanism suitable for power-constrained networks such as Wireless Sensor Networks (WSN) and LPWAN. However, the limitation of BYka is that it does not provide a session key mechanism. As mentioned in [24], if the session key is required, the nodes in the networks will have to use the generated key from BYka to encrypt the session key and transfer it to other nodes. This increases the processing and adds transmissions for nodes in the network, and the session keys are to be sent over the air. The proposed extension to BYka (extended BYka/proposed mechanism) is for LPWAN that takes advantage of the star topology, where there are computationally efficient devices on the internet-facing end of the network. The processing is offloaded from end nodes to these efficient devices to minimize the processing at the end node. In the proposed mechanism, a centralized key management server is utilized for all session key management operations. The server stores all the master keys and uses the operations from Equation (2) to calculate the private keys for each node. Figure 2 shows the architecture of the proposed session key infrastructure, including each network entity.

Seed Management for Session keys: Assuming that E = {ID_1_, ID_2_, ID_3_, …, ID_n_} is a set consisting of all the nodes in the network. The key generation server maintains a set U consisting of unused Node IDs such that U ∩ E = Φ.

**Step 1**: The server chooses a random ID from U.

**Step 2**: The chosen random ID will be broadcast in the network for pairwise key generation using BYka.

**Step 3**: The used ID will be removed from U and put in a different set of used IDs.

For every session, a new random ID is chosen from U to broadcast for a new pairwise key generation. The randomization of ID at one end causes the change in node pair for every session. Hence, for every session, the pairwise key will be different. If a new node joins the network such that U ∩ E ≠ Φ, that particular Node ID is simply removed from U to maintain the condition of U ∩ E = Φ.

**Figure 3:**
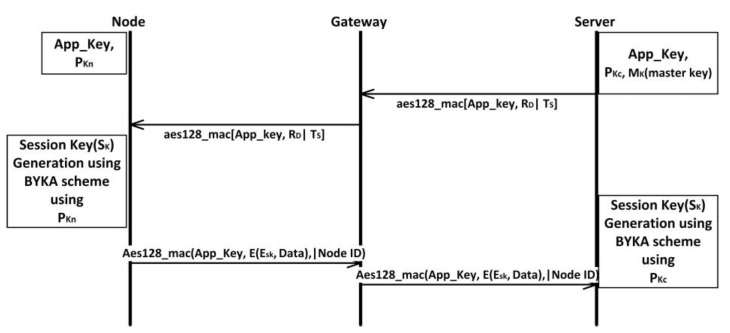
Sequence Diagram for the Session Key Generation.

As the transmission of data from the server to the node is wireless, the network is vulnerable to replay of the captured packet and data modification attack. To avoid such attacks, some additional measures are added to the key exchange mechanisms. Figure 3 shows the sequence of operations performed for session key generation.

Infrastructure setup for the Server:
The server stores the master key M;

E = {ID_1_, ID_2_, ID_3_, …, ID_n_}

where E is the set of IDs of the nodes in the network

U is a set of random IDs such that U ∩ E = Φ.

Infrastructure setup for the Node:

Nodes are pre-loaded with an App Key and private keys.

The App Key is shared between servers and nodes and is unique to all the applications in the network. The private key is calculated using the Master Key by the server.

**Step 1**: The server initiates the communication with a message a Random ID (*R_D_*) taken from the set U, a timestamp *T_S_*, and AES-128 message authentication code (MAC) calculated with App Key shared by both node and the server.

*Server Message = AES-128 MAC*[*App Key, R_D_|T_S_*]

**Step 2**: The gateway forwards the message to the end node.

**Step 3**: Upon receiving the message from the gateway, the node verifies the MAC to confirm the integrity of the message.

**Step 4**: *R_D_* is used to calculate a public key of the sender using Equation (1).

**Step 5**: The node uses its private key to calculate the session key *S_k_* using Equation (3).

**Step 6**: The node uses *S_k_* to encrypt the data with AES-128, appends the encrypted data with its ID, calculates the MAC using the App Key, and sends it to the server.

*Node Message = AES-128 MAC(App Key, E(E_sk_, Data)|Node ID)*

**Step 7**: Upon receiving the message from the node, the MAC of the message is verified.

**Step 8**: If the integrity of the message is intact, the Node ID of the node is used to generate a public key using Equation (1).

**Step 9**: The *R_D_* sent in step 1 is used to generate a private key from the server-side by using Equations (1) and (2).

**Step 10**: The private key generated in step 9 and public key from step 8 is used to generate session key *S_k_* (same as node side) using Equation (4) which is used to decrypt the message from the node.

**Step 11**: For every session, a unique value from *U* is selected or chosen to generate a new pair which results in the generation of a new session key every time.

The operations for key calculations performed at both the node and the server-side are shown below

Node A (End Node):

for *i*, *k* = 1, …, *n* and *j* = 1, …, N

(5) $$\begin{array}{c} s_{Bk}\;=\;R_{D}\;+\;k-1\\ V_{Bk}^{T}\;=\;[1,\;s_{Bk},\;\ldots s_{Bk}^{m-1}]\\ R_{Aijk}\;=\;K_{Aij}V_{Bk}\;=\;(V_{Ai}^{T}M_{j})V_{Bk}(mod\;p) \end{array}$$

Node B (Server):

for *i*, *k* = 1, …, *n* and *j* = 1, …, N

(6) $$\begin{array}{c} s_{Ak}\;=\;ID_{A}\;+\;k-1\\ V_{Ak}^{T}\;=\;[1,\;s_{Ak},\;\ldots s_{Ak}^{m-1}]\\ s_{Bk}\;=\;R_{D}\;+\;k-1\\ V_{Bk}^{T}\;=\;[1,\;s_{Bk},\;\ldots s_{Bk}^{m-1}]\\ K_{Bij}\;=\;V_{Bki}^{T}M_{j}(mod\;p)\\ R_{Bijk}\;=\;K_{Bij}V_{Ak}\;=\;(V_{Bi}^{T}M_{j})V_{Ak}(mod\;p) \end{array}$$

### 3.3. Features of the Extended BYka Scheme

As discussed in Section 3.2, the extended BYka scheme takes advantage of the star topology of LPWAN. BYka was designed for wireless sensor networks; hence it has to use static keys stored in the node to generate the secret key during key agreement. However, LPWAN is not restricted to this limitation. There are a number of advantages of extended BYka for LPWANs utilizing star topology discussed as follows:

**Prevention Against Replay Attacks**: In wireless transmission technologies, there are high chances of adversaries using packet sniffing as a tool to pose an attack. The attacker uses a transceiver to capture the packets that travel between the nodes and the gateways or vice versa. The possibility of injecting these captured packets back into the network by the attackers breaches the network’s integrity and the data’s authenticity. These types of attacks are called replay attacks, where captured packets are replayed in the network. Current LPWAN techniques provide various mechanisms to counter the possibility of replay attacks in the networks by using frame counters or nonces. However, when it comes to session key mechanisms, LoRaWAN is the only LPWAN technology that provides it. LoRaWAN uses join-request to initiate the session keys from end nodes, and the end nodes receive join-accept messages from servers as a response. LoRaWAN uses DevNonce to avoid the replay of join-request messages. However, the join-accept messages do not have a Nonce when sent from the server, creating a possibility of a replay of join-accept messages in the network [12]. The replay of join-accept messages in the network can cause an end node to trust a fake gateway.

To prevent replay attacks, in the proposed session key mechanism, the session initiation packets from the central server carry a timestamp that identifies the age of the packets in the network. The nodes can compare the timestamps with their clocks and decide whether the packet is fresh or is being replaced by an attacker. As the timestamps are included in MAC calculations, the attackers cannot change it if they capture the packet.

**Robust Session Control:** The BYka scheme is designed for wireless sensor networks where nodes have to communicate with each other to relay data to the gateway node. The originator of the data initiates the key exchange process with whom it wants to communicate. In a similar manner, every node initiates the key agreement process with the successor node in the routing path as the data moves towards the gateway. However, the proposed mechanism targets the LPWANs where the gateway directly receives data from the end nodes and is aware of the node scheduling. Thus, the gateway initiates the sessions with the end nodes. As the central servers are aware of the applications running on the nodes in the network, the sessions can be personalized based on the application’s security requirements. The centralized session control provides the ability to consider multiple factors based on the status of the end nodes (application, remaining energy, transmission frequency, location). The central server can manage the session lengths based on the remaining energy of the node and applications running on the node to optimize the energy consumption of the end node.

**Lightweight:** As BYka targets to provide a key agreement mechanism in sensor networks for data encryption, it uses scalar multiplications on a matrix using comparatively small prime numbers for key calculation. The simple operations of BYka and the reduced number of transmissions in the proposed framework are achieved by shifting the extensive calculations towards the central server, making the proposed key exchange framework suitable for constrained networks.

**Node Authentication:** The proposed session key mechanism enables the automatic authentication of the end node without additional transmission because only the nodes pre-loaded with private keys from a central server can generate the same session key as the server on receiving the public information.

**Minimal Transmissions**: Considering the limitations of the end nodes for limited energy availability, the proposed session key mechanism shifts most of the session key responsibility towards the server. Unlike the LoRaWAN session key mechanism, the proposed mechanism makes the central server responsible for initiating the session keys, considering the sleep cycles of the nodes running different applications. Hence, the nodes only have to receive the session key information, generate the session keys, and use the session keys for data encryption prior to sending the data packet. The central server is responsible for validating the data authenticity at its end by using the session key generated at its end. In case the server finds any validation failure, it simply discards the packets. Hence, the transmission from the end nodes is minimal and can maximize the lifetime of the nodes while using the session key mechanism.

## 4. Experimentation and Analysis

In a constrained environment of LPWANs, every operation performed at the end node can shorten the node lifetime. Considering the limitations LPWANs, the proposed key exchange mechanism aims to minimize the operations on the end nodes of the network.

### 4.1. Correctness of The Key Exchange Mechanism

**Figure 4:**
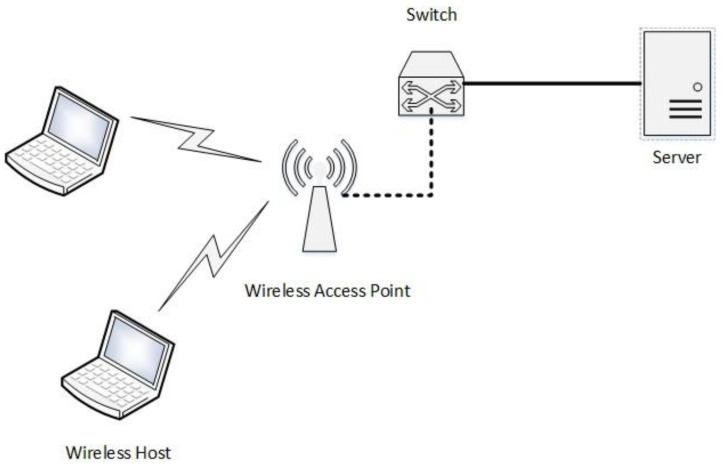
Mininet-WiFi Experiment Topology.

The key exchange mechanism uses mathematical calculations at the server and the end nodes in the network. The session key is calculated by performing mathematical operations on the public information transferred by the server to the end node. To validate the correctness of the key exchange mechanism, Mininet-WiFi [26] is used as an emulator. The proposed key exchange mechanism is written as a python script for both the end node and server node to run on their sides. Figure 4 shows the Mininet-WiFi topology created to simulate a wireless network for key exchange experimentation.

**Figure 5:**
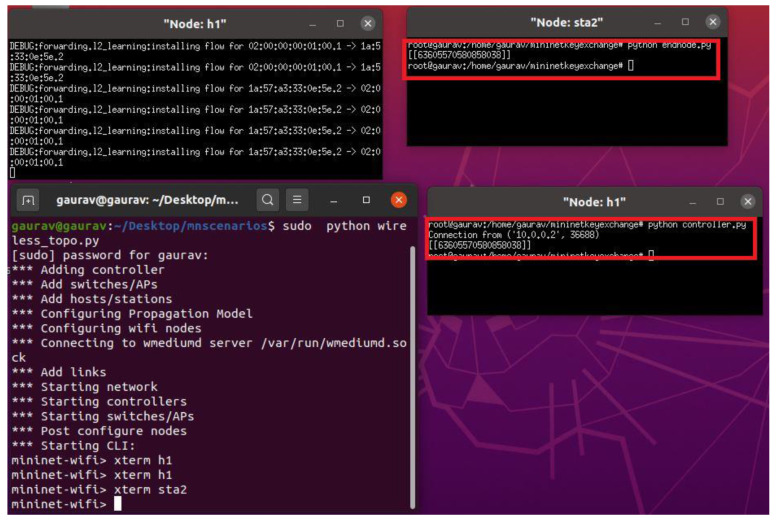
Key Exchange Verification in Mininet-WiFi.

Figure 5 shows the implementation of the session key mechanism on Mininet-Wi-Fi in the scenario shown in Figure 4. It shows a successful key exchange between the server and the end node where both the server node: h1 and the end node: sta1 come to a common key agreement that can be used for encryption by the nodes in the network for confidential data transmission.

### 4.2. Security Analysis

The proposed key exchange mechanisms have various aspects that require verification of effective operations. One of the aspects is secure session key agreement without losing the integrity of the public key information being transmitted between the server and the nodes over the air. There are several mechanisms to verify the security of security protocols. One of the well-known techniques is BAN logic [27]. However, considering the manual process and limitations of these mechanisms, the security verification tool Scyther [28] is used.

Scyther is a well-known tool for the analysis of security protocols. It is assumed that cryptographic functions are perfect, meaning the adversary learns nothing from encrypted messages unless they have a decryption key. The tool is used to find loopholes in the models used for investigating security protocols. The protocol is formulated in the form of “roles” and “events”. The “roles” are designed based on the entity’s knowledge and operations in the protocol model. “Event” describes the events of sending and receiving of the data. Based on the operations, the roles make claims regarding secrecy, integrity, and authenticity. These claims are verified over several threat models defined in Scyther. Based on the analysis, possible attacks are shown on the security protocol model. The security model shown in Figure 3 is implemented using the Scyther input language to verify the secrecy of the session key, sever, and device agreement against possible attack models. Table 1 shows the results of the security analysis of the flow shown in Figure 3. The proposed mechanism verifies all the claims (details of claims in Scyther can be found in [28]) and that no possible attacks are successful in Scyther’s attack model.

The proposed session key mechanism uses the BYka scheme as a cryptographic primitive and inherits the node capture threshold to calculate the master key. As each node has Nn private keys, to construct the N (m × m) master keys, the adversary needs to capture $\frac{m}{n}$ nodes. However, the captured threshold is only effective if each captured private key can be correctly associated with the public key and the master key used to calculate it [24].

### 4.3. Computational Analysis

In addition to the security model’s semantic analysis, its operational overhead must be minimized for the constrained nodes in LPWAN networks. Implementing computationally intensive security mechanisms can shorten the end node lifetime causing disruptions in the network functionality.

The proposed security model is designed to minimize the number of transmissions and processing overhead for end nodes to minimize the node’s energy consumption. To examine the energy consumption of the proposed scheme, we have created an energy model based on the energy consumption of various operations such as data transmission, data reception, data encryption, and BYka key calculation.

Considering that the energy required to transmit the data is ETX, to receive the data ERX, BYka calculation EBK, for AES MAC calculation EMAC, and for AES encryption EAES. As shown in Figure 3, for every transmission, the end node performs two operations before transmitting data to the server. Hence, we can calculate the energy of operation (*E_OP_*) for each transmission as follows:

*E_OP_ = SUM(E_RX_, E_BK_, E_MAC_, E_AES_)* (7)

Equation (7) considers every transmission as a different session. However, if the length of the sessions is made based on the number of transmissions *m* for the total *n* number of transmissions. The node energy consumption will be reduced and calculated as follows:

(8) $$E_{OP}=\;(\Sigma_{i=1}^{n}\;\;E_{RXi}+E_{MACi}+E_{AESi})\;+\;\Sigma_{j=1}^{n/m}\;E_{BKj}$$

### 4.4. Simulation Parameters

In the experiments, the standard implementation of the energy model of LoRaWAN in Network Simulator 3 (NS3) [29] is modified to study the effects of the session key operations on the end nodes. The standard NS3 LoRaWAN energy model implementation only considers energy consumption of data transmission and reception. The modified energy model adds the energy consumption of cryptographic and BYka operations to the existing LoRaWAN NS3 implementation. The energy model for AES encryption and MAC operations is derived from the energy consumption of cryptographic algorithms discussed in [30]; the energy consumption for BYka key agreement is based on its sensor node implementation discussed in [24].

The simulation is designed to analyze the energy consumption and node lifetime in a LoRaWAN network using a scenario with a single gateway and end node. The end node transmits a packet every five seconds to the gateway while consuming energy according to Equation (7). Table 2 shows the summary of simulation parameters used in NS3.

**Figure 6:**
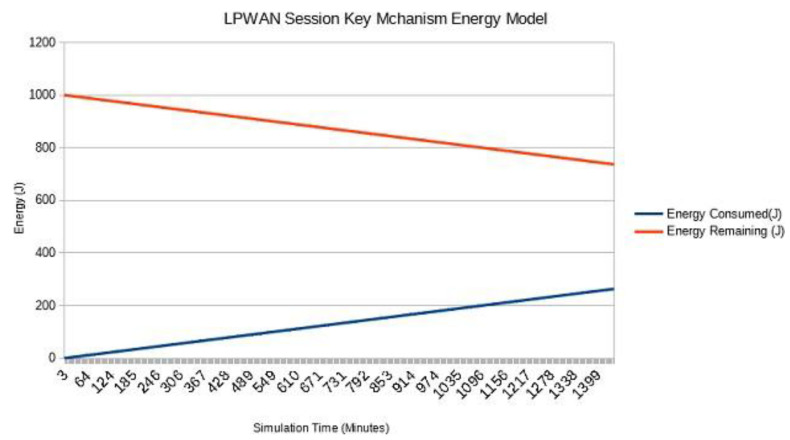
Energy Consumption of the LPWAN Session Key Mechanism.

As the proposed mechanism focuses majorly on LPWANs that are used for low data rate applications [31], the experiments are performed on lower data rates to address the LPWAN IoT applications. Figure 6 shows the energy consumption of the end node over twenty-four hours of transmissions with a data rate of 12 packets per minute. Our experiments considered every transmission as a session, and different key generation operations are performed for every transmission. The number of packets for each session can be decreased, and the overhead of session key generation can be reduced on the end nodes as per security requirements. However, in this experiment, we considered the worst-case scenario of a maximum overhead on end nodes. If the energy consumption of the end node remains the same as shown in Figure 6, the node can remain functional for one and a half months with 10,000 J energy which is equivalent to an 850 mAh battery with a 3-volt supply.

To further investigate the impact of overhead caused by the proposed session key mechanism, additional simulations are performed on the NS3 simulator with a simulation time of 30 days to compare the energy consumption with and without the session key mechanism on the LoRaWAN protocol. As the NS3 energy model for LoRaWAN did not consider the energy consumption of encryption and MAC calculations, it was added based on results [30]. Table 3 shows the comparison of LoRaWAN protocol with and without session key mechanism based on different data rates on end nodes. With high data rates on the end nodes, the impact of the session key mechanism is much higher if we change the session key for each packet. However, as the data rate decreases and the frequency of the session decreases, the difference between power consumption with and without the session key mechanism narrows. It can be observed in Table 3 that the power consumption with a session key in the case of 1 packet/minute is more than double the power consumption without a session key mechanism. The difference between them comes down to almost fifty percent in the case of the data rate 1 packet/30 min. For applications running 1 packet/6 h, the difference is only one Joule over a month. Furthermore, as we move on to the cases of even lower data rates, the difference between the power consumption in both cases becomes negligible, implying that we can achieve a session key mechanism with negligible overhead for low data rate applications. Some of such applications that need infrequent low-speed communications are trucking and logistics, smart parking, and remote monitoring of water or gas pipes [32].

IoT nodes are usually designed to last longer while sending data with low data rates (depending on the applications running on the nodes). Considering the impact of the session key mechanism in high data rates, changing sessions for each packet may not be ideal. However, given that the session lengths are regulated, the impact of the session key mechanism can be minimized, as shown by Equation (8). Thus, making it suitable to run on applications with high data rates. On the other hand, applications with lower data rates can afford to change the session with each packet without having a severe impact on the power consumption of the end nodes. Resulting in the end nodes having a similar lifetime as standard LPWAN protocols while utilizing the session key mechanism.

### 4.5. Comparison with Existing Session Key Mechanisms

The experiments show that the proposed session key mechanism provides protection against various attacks on the network. The correctness of the session key mechanism is verified by implementing and demonstrating it on the Mininet-WiFi emulator. The proposed mechanism considers the vulnerability of the LoRaWAN join procedure to possible replay attacks of join-accept messages and uses timestamps to avoid replays of packets being sent from the server for the session key mechanism. Also, unlike LoRaWAN, the key exchange is initiated by the server, not the node, providing more control over the session management and key exchange by using broadcast messages. Besides, as the node does not initiate the key exchange, the proposed mechanism further reduces the number of transmissions from the end nodes for session key generation.

Table 4 shows a comparison of the number of transceiver operations required by some existing and the proposed session key mechanisms in LPWAN. The table shows that approach in [21] and standard LoRaWAN [4] requires two transceiver operations for the nodes to generate the session keys. The session key mechanism in [15] requires a much higher of five transceiver operations to generate the session keys. On the other hand, the proposed session key mechanism only requires a single transceiver operation which is half of the standard LoRaWAN and approach in [21], and one-fifth of the session key mechanism in [15]. The transceiver operation is one of the most expensive operations in wireless networks [23]; by minimizing it to a single transmission of the session keys, the proposed mechanisms will have a significant impact on extending the node lifetime in an IoT network.

In addition to minimal transmission requirements, the proposed mechanism also allows flexible control of session lengths based on network state. As the servers initiate the key exchanges, they can be programmed to consider factors such as the node’s remaining energy and applications running on nodes before initiating the session key generation.

## 5. Conclusions

LPWAN is one of the most prominent transmission technologies used in IoT applications. The low-powered long-range transmission support of LPWAN makes it viable for use in scenarios where nodes are deployed in an adverse situation and require low-speed infrequent communications. However, apart from the successful transmission of data to central servers, data security is equally challenging in LPWAN.

The current LPWAN technologies focus more on data transmission while using basic security mechanisms in the networks. As discussed in the literature, the current security mechanisms lack several security fronts, such as a lack of session key mechanisms and static key use for the whole lifetime of a node. LoRaWAN provides session key generation, but the key generation mechanism is vulnerable to various attacks. In this research, a lightweight session key mechanism initiated by a central server is proposed.

The proposed session key mechanism minimizes the number of transmissions required from the end node for session key generation. The session key mechanisms ensure the authentication of nodes and secure session key generation using public data. The proposed mechanism is implemented in python on a Mininet-WiFi emulator to verify its correctness. The semantic analysis of the session key mechanism shows that it can maintain the authenticity and the security of the session key with no possible attack being successful, as shown in the Scyther tool’s attack model. In addition, the energy model of the security mechanism is validated using the NS3 simulator.

The proposed session key provides a mechanism against replay attacks using timestamps during key exchanges. Also, as the servers initiate the sessions, servers can be programmed to manage the sessions based on the application requirements of the network devices. As the proposed mechanism uses a different key for each node, the impact of a node being compromised in case of a physical attack does not impact the entire network.

The current implementation focuses on LPWAN’s LoRaWAN standard, where the proposed session key mechanism uses a centralized server for a key generation that might introduce a single point of failure. To address the single point of failure, the utilization of redundant servers as a backup should be considered in future implementations. Furthermore, we also plan to focus on implementing the session key mechanisms on various LPWAN testbeds such as Sigfox and NB-IoT to demonstrate the feasibility of the session key mechanism on other LPWAN standards.

## Figures and Tables

**Table 1:** Security Analysis Results of the LPWAN Session Key Mechanism.

Claim	Status	Attack Patterns
Network Entity: Server		
Alive	OK Verified	No Attacks
Weakagree	OK Verified	No Attacks
Niagree	OK Verified	No Attacks
Nisynch	OK Verified	No Attacks
SKR(Rd)k(Dev, Srv)	OK Verified	No Attacks
	Network Entity: End Node	
Alive	OK Verified	No Attacks
Weakagree	OK Verified	No Attacks
Niagree	OK Verified	No Attacks
Nisynch	OK Verified	No Attacks
SKR(Rd)k(Dev, Srv)	OK Verified	No Attacks

**Table 2:** Simulation Parameters.

Parameters	Values
Simulation Time	24 h
Initial Node Energy	10,000 J
Supply Voltage	3.3 V
Current for Packet Transmission	0.028 A
Current for Packet Reception	0.0112 A
Number of Gateways	1
Number of Nodes	1
Data Rate	12 Packets/Minute

**Table 3:** LoRaWAN Energy Consumption Analysis.

Data Rate	LoRaWAN Energy Consumption with Session Key Mechanism (J)	LoRaWAN Energy Consumption without Session Key Mechanism (J)
1 packet/minute	692.16 J	272.122
1 packet/30 min	35.4716	21.4703
1 packet/hour	24.1464	17.1457
1 packet/6 h	14.6801	13.5133
1 packet/12 h	13.6992	13.1158
1 packet/24 h	13.1527	12.8611
1 packet/7 days	10.992	10.9531
1 packet/15 days	10.6205	10.6001

**Table 4:** Transceiver Operation Comparison with Existing Session Key Mechanisms.

Session Key Mechanisms	Number of Transceiver Operations
An enhanced key management scheme for LoRaWAN [15]	5
A Dual Key-Based Activation Scheme for Secure LoRaWAN [21]	2
LoRaWAN OTA Activation [4]	2
Proposed Session Key Mechanism	1

## Data Availability

The data presented in this study are available on request from the corresponding author.
